# Altered Spontaneous Brain Activity Patterns and Functional Connectivity in Adults With Intermittent Exotropia: A Resting-State fMRI Study

**DOI:** 10.3389/fnins.2021.746882

**Published:** 2021-10-29

**Authors:** Xueying He, Jie Hong, Qian Wang, Yanan Guo, Ting Li, Xiaoxia Qu, Jing Liu, Wei Li, Lirong Zhang, Jing Fu, Zhaohui Liu

**Affiliations:** ^1^Department of Radiology, Beijing Tongren Hospital, Capital Medical University, Beijing, China; ^2^Department of Ophthalmology, Beijing Tongren Hospital, Capital Medical University, Beijing, China

**Keywords:** intermittent exotropia, functional connectivity, amplitude of low-frequency fluctuations, resting-state functional MRI, spontaneous activity

## Abstract

The purpose of this study is to investigate brain functional changes in patients with intermittent exotropia (IXT) by analyzing the amplitude of low-frequency fluctuation (ALFF) of brain activity and functional connectivity (FC) using resting-state functional magnetic resonance imaging (rs-fMRI). There were 26 IXT patients and 22 age-, sex-, education-, and handedness-matched healthy controls (HCs) enrolled who underwent rs-fMRI. The ALFF, fractional ALFF (fALFF) values in the slow 4 and slow 5 bands, and FC values were calculated and compared. The correlations between ALFF/fALFF values in discrepant brain regions and clinical features were evaluated. Compared with HCs, ALFF/fALFF values were significantly increased in the right angular gyrus (ANG), supramarginal gyrus (SMG), inferior parietal lobule (IPL), precentral gyrus (PreCG), and the bilateral inferior frontal gyri (IFG), and decreased in the right precuneus gyrus (PCUN), left middle occipital gyrus (MOG), and postcentral gyrus (PoCG) in IXT patients. The Newcastle Control Test score was negatively correlated with ALFF values in the right IFG (*r* = −0.738, *p* < 0.001). The duration of IXT was negatively correlated with ALFF values in the right ANG (*r* = −0.457, *p* = 0.049). Widespread increases in FC were observed between brain regions, mainly including the right cuneus (CUN), left superior parietal lobule (SPL), right rolandic operculum (ROL), left middle temporal gyrus (MTG), left IFG, left median cingulate gyrus (DCG), left PoCG, right PreCG, and left paracentral gyrus (PCL) in patients with IXT. No decreased FC was observed. Patients with IXT exhibited aberrant intrinsic brain activities and FC in vision- and eye movement-related brain regions, which extend current understanding of the neuropathological mechanisms underlying visual and oculomotor impairments in IXT patients.

## Introduction

Comitant strabismus is a group of developmental diseases involving ocular motility impairment (Ouyang et al., 2017; Li et al., 2018). Intermittent exotropia (IXT) is the most common subtype of comitant strabismus, accounting for 58% of cases, with a prevalence ranging from 0.12 to 3.9% of the population worldwide (Chia et al., 2010; McKean-Cowdin et al., 2013; Fu et al., 2014; Pan et al., 2016). IXT manifests as intermittent squinting outward during distance fixation or inattention, which always gives rise to psychological problems and impaired social interaction (Brodsky and Jung, 2015; Yang et al., 2016; Zhu et al., 2018).

The precise pathological mechanisms underlying IXT remain unclear. Many authors proposed that IXT is caused by defective binocular fusion in the brain (Guyton, 2006; Brodsky and Jung, 2015). Abnormal fusion function leads to an inability to form normal stereoscopic vision. Li et al. (2016) found abnormal brain activity in binocular fusion-related cortices in IXT patients using task-based functional magnetic resonance imaging (fMRI). However, compared with task-fMRI, resting-state functional magnetic resonance imaging (rs-fMRI) requires minimal patient compliance, avoids potential performance confounders associated with cognitive activation paradigms in task-fMRI research, and is relatively easy to implement in clinical studies (Turner, 2013). Yang et al. (2014) and Tan et al. (2016) confirmed that the abnormal brain activity was exhibited in infantile esotropia and congenital comitant strabismus using rs-fMRI, which cannot totally represent the brain functional changes of IXT. Unfortunately, no study about IXT, a subtype of comitant exotropia, explores the spontaneous neural activity changes of attenuation of spontaneous blood oxygen level dependent (BOLD) fluctuations using rs-fMRI at present.

Analyses of the amplitude of low-frequency fluctuations (ALFF) and functional connectivity (FC) are two important methods used in rs-fMRI studies. ALFF is an rs-fMRI analysis technique used to measure spontaneous fluctuations of neural activities in BOLD signals, which has been reported to be a useful parameter by many authors (Logothetis et al., 2001; Biswal, 2012). Fractional ALFF (fALFF) is an index reflecting the relative contribution of specific low-frequency oscillations to the entire frequency range (Zhou et al., 2013). In previous rs-fMRI studies, the frequency band of 0.01 to 0.1 Hz most commonly revealed spontaneous oscillation activities, but lower frequency bands can better reflect local neural activity and reduce the influence of distant neural activity. The slow 4 (0.027–0.073 Hz) band and slow 5 (0.01–0.027 Hz) band in fALFF were deemed to be more sensitive and specific frequency bands for intrinsic brain activity to analyze alterations in neural activity (Han et al., 2012; Zhou et al., 2013). A combination of the two parameters can be used to acquire more information about the brain in IXT patients and verify abnormal functional activity (Li et al., 2014). FC analysis is an effective method with which to estimate spontaneous functional activity and it measures temporal relevance between the BOLD signals of two brain areas that occur in turn (Tomasi and Volkow, 2010). Analyses of ALFF, fALFF, and FC simultaneously allow researchers to explore the synchronization between brain regions and the activation of each brain region.

In this study, we investigated the ALFF, fALFF, and FC value changes of the whole brain using rs-fMRI in IXT patients and explored the correlation between the ALFF/fALFF value changes and clinical features, including the duration and severity of IXT. In accord with previous studies of strabismus, we hypothesized that IXT patients would exhibit aberrant intrinsic spontaneous brain activity and FC.

## Materials and Methods

### Subjects

A total of 26 IXT patients (14 male individuals, 12 female individuals, age = 28.23 ± 8.14 years) were enrolled in this study. In addition, we recruited 24 healthy control subjects (HCs) (14 male individuals, 10 female individuals, age = 28.82 ± 6.48 years) from the local community, matched with IXT patients in terms of gender, age, education, and right or left handedness. All participants underwent MR examination and a series of ophthalmological examinations, including measurement of best corrected visual acuity, fundus examination, synoptophore, alternate cover test, and the Newcastle Control Test (NCT). This study was approved by the medical research ethics committee and institutional review board of Capital Medical University, Beijing Tongren Hospital, and written informed consent was obtained from all participants.

The IXT patients were recruited based on the following criteria: (1) evidence of IXT on the basis of history and clinical examination (loss of stereoscopic vision detected by synoptophore and/or deflection time accounted for more than half of waking time, and exotropia deviation < −15Δ), (2) best-corrected visual acuity (VA) ≥1.0, (3) adults over 18 years old, (4) no ongoing amblyopia treatment, and (5) ability to understand and cooperate with inspection, and voluntarily provide written informed consent. The inclusion criteria of HCs included the (2)–(5) of the above standards and free of any ocular diseases. The exclusion criteria included: (1) other kinds of strabismus, such as constant exotropia, esotropia, or incomitant strabismus, (2) ocular diseases (e.g., amblyopia, cataract, glaucoma, optic neuritis, and macular degeneration), (3) accompanied by vertical strabismus, (4) previous eye surgery history, (5) history of diseases involving psychiatric, cardiovascular, or cerebral disease, (6) drug or alcohol addiction, and (7) contraindication for MRI examination (treatable or accidental magnetizable metal in cardiac pacemaker or prosthesis, or previous head or spinal trauma requiring neurosurgery), (8) head motion larger than 2 mm maximum displacement in any direction or an angular rotation greater than 2° throughout the scan. According to these criteria, 26 IXT patients and 22 HCs were enrolled in this study.

### Magnetic Resonance Imaging Data Acquisition

Images were acquired using a 3.0 T MRI scanner (Discovery MR750; General Electric, Milwaukee, WI, United States). A matched eight-channel phased array coil was used with earplugs and foam padding to reduce scanner noise and head motion. The images were parallel to the anterior commissure (AC)-posterior commissure (PC) line and covered the whole brain. Resting-state fMRI was obtained using an echo planar imaging (EPI) pulse sequence with the following parameters: repetition time (TR)/echo time (TE) = 2000/35 ms, flip angle = 90°, field of view (FOV) = 240 mm × 240 mm, matrix = 64 × 64, voxel size = 3.75 mm × 3.75 mm × 4.0 mm, and 180 time points. Axial slices were obtained with 5 mm thickness. The pulse duration of each fMRI session was 400 s. The three-dimensional brain volume (3D-BRAVO) sequences were used to acquire high-resolution structural images (TR = 8.16 ms, TE = 3.18 ms, TI = 450 ms, matrix = 256 × 256, FOV = 240 mm × 240 mm, slice thickness = 1.0 mm without gap, flip angle = 12°, gap = 0 mm, 188 slices, and voxel size = 1 mm × 1 mm × 1 mm). The total scan duration of the 3-dimensional brain volume sequence was 259 s. During scanning, subjects were asked to remain motionless, to stay awake, and not to think of anything specific until the scan was over.

### Data Preprocessing

Preprocessing was carried out using Data Processing Assistant for Resting-State fMRI (DPARSF 4.2; State Key Laboratory of Cognitive Neuroscience and Learning, Beijing Normal University, Beijing, China^1^) and CONN functional connectivity toolbox (Whitfield-Gabrieli and Nieto-Castanon, 2012) based on Statistical Parametric Mapping (SPM12)^2^ running under MATLAB R2013b (The MathWorks, Natick, United States). The first 10 volumes were removed to allow participants to adapt to the scanning noise and the signal equilibrium was measured after converting DICOM files to NIFTI images. Slice timing, head motion correction, spatial normalization to the Montreal Neurological Institute (MNI) template (resampling voxel size = 3 mm × 3 mm × 3 mm) were then performed. We used a linear regression process to remove the effects of head motion and other possible sources of artifacts: (1) six motion parameters, (2) whole-brain signal averaged over the entire brain, (3) white matter signal, and (4) cerebrospinal fluid signal. In addition, the linear trend of time courses was removed. Preprocessing of voxel-based morphometry (VBM) was carried out using DPARSF 4.2. The imaging of 3D-BROVA was converted from DICOM files to NIFTI images. Then segmentation and spatial normalization were performed to extract the gray matter volume (GMV). After that the images were smoothed by a Gaussian kernel (full width at half-maximum of 8 mm).

### Voxel-Wise Amplitude of Low-Frequency Fluctuations/Fractional Amplitude of Low-Frequency Fluctuation Analysis

DPARSF 4.2 was performed to calculate the ALFF and fALFF values. Briefly, the time courses were first transformed to the frequency domain using Fast Fourier Transform (FFT) (parameters: FFT length = shortest, taper percent = 0). The square root of the power spectrum obtained by FFT was computed, then averaged across 0.01–0.08 Hz at each voxel, which was taken as ALFF. Fractional ALFF is the fraction of amplitude of low-frequency fluctuation in a given frequency band over the entire frequency range. The low-frequency range was further disassembled into a slow 4 (0.027–0.073 Hz) and a slow 5 (0.01–0.027 Hz) band for the BOLD signal, to make the data more robust against physiological noise. The fALFF values were then calculated for slow 4 and slow 5 bands. The ALFF/fALFF value for each voxel was divided by the global mean ALFF/fALFF value for each participant to reduce the global effects of variability. Finally, the ALFF/fALFF images were smoothed by a Gaussian kernel (full width at half-maximum of 6 mm).

### Voxel-Wise Functional Connectivity Analysis

Before FC analysis, a temporal bandpass filter (0.01–0.08 Hz) was used to minimize the effects of low frequency drift and high frequency noise. Then FC analysis was performed using the CONN toolbox. Based on the ALFF result, the region that showed significant difference between the IXT patient and HCs was defined as the region of interest (ROI) using DPARSF 4.2. After that, we performed a voxel-wise FC analysis by computing the temporal correlation between the mean time series of the ROI and the time series of each voxel within the brain. The data were transformed using Fisher r-to-z transformation to improve normality of the correlation coefficients. Meanwhile, the FC images were smoothed by a Gaussian kernel (full width at half-maximum of 6 mm).

### Validation Analysis

Given that GMV atrophy may affect the ALFF/fALFF and FC changes, we analyzed intergroup differences in both GMV to evaluate whether any significant gray matter (GM) changes were significant in IXT patients. We compared GMV between the IXT and HC groups using a two-sample *t*-test in SPSS and applied a voxel-wise comparison to identify structures that differed significantly in volume between the two groups. There was no report about GM atrophy previously in IXT patients, however, we investigated the ALFF/fALFF comparisons with GMV as an additional covariate to exclude the effect of GM atrophy.

### Statistical Analysis

To examine the demographic data, clinical features, and head motion parameters, a two-sample *t*-test was used for normally distributed data, and the Mann–Whitney U test was used for non-normally distributed data. Statistical significance was set at *p* < 0.05. A one-sample *t*-test (*p* < 0.05, Gaussian random field (GRF)-corrected) was used to extract ALFF results across subjects within each group. A two-sample *t*-test was used to compare the ALFF and fALFF values for the slow 4 and slow 5 bands differences between groups, while age, gender, and education were included as covariates. The statistical threshold was set at a voxel level of *p* < 0.001 and a cluster level of *p* < 0.05, GRF-corrected. Pearson correlation analysis was performed using SPSS to explore the relationships between mean ALFF/fALFF and mean FC values of discrepant brain regions and NCT scores, IXT duration in the patient group with a *p* < 0.05. A two-sample, two-tailed *t*-test was performed to compare the FC maps between IXT patients and HCs, while age, gender, and education were included as covariates. The statistical threshold was set at a voxel level of *p* < 0.001 and a cluster level of *p* < 0.05, GRF-corrected.

## Results

### Demographics and Clinical Features

Clinical characteristics of enrolled 26 IXT patients and 22 HCs are listed in Table 1. There was no significant difference in age (*p* = 0.786), sex (*p* = 0.572), education (*p* = 0.726), handedness (*p* > 0.999), and the best-corrected VA(R/L) (right: *p* = 0.160; left: *p* = 0.094). Head motion parameters did not differ significantly between groups (*p* = 0.231).

**TABLE 1:** Demographics and clinical measurements of IXT patients and HCs.

	**IXT (*n* = 26)**	**HC (*n* = 22)**	**t-value**	**P-value**
Sex, male/female	14/12	10/12	0.569	0.572*
Age (years)	28.23 ± 8.14	28.82 ± 6.48	−0.273	0.786^†^
Handedness	26R	22R	N/A	>0.999*
Education (years)	15.38 ± 2.93	16.59 ± 2.72	−1.484	0.726^†^
Duration (years)	10.33 ± 9.12	N/A	N/A	N/A
Newcastle control test	5.25 ± 1.87	N/A	N/A	N/A
The best-corrected VA (R)	0.98 ± 0.11	1.02 ± 0.07	−1.433	0.160^†^
The best-corrected VA (L)	0.98 ± 0.11	1.04 ± 0.13	−1.720	0.094^†^
Prism diopter/near	−73.26 ± 34.43	N/A	N/A	N/A
Prism diopter/distance	−69.57 ± 35.32	N/A	N/A	N/A
Mean fd-power	0.11 ± 0.04	0.10 ± 0.03	1.213	0.231^†^

*Data are presented as mean ± SD; HC, healthy control; IXT, intermittent exotropia; N/A, not applicable; VA, visual acuity; R, right; L, left.*

*Chi-square test.

*^†^Two-sample *t*-test.*

### Voxel-Wise Amplitude of Low-Frequency Fluctuations and Fractional Amplitude of Low-Frequency Fluctuation Differences

**FIGURE 1:**
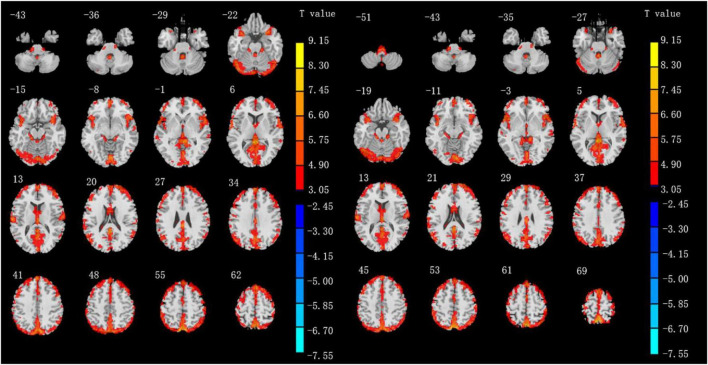
Results of ALFF values of IXT patients **(left)** and HCs **(right)** in the rs-fMRI using one-sample *t*-test. (*P* < 0.05, GRF correction).

Altered ALFF values across all subjects in the two groups during the resting state are shown in Figure 1. The general trend of regional brain activity was similar between the two groups and some regions exhibited significantly higher ALFF values than other brain regions during the resting state, including the bilateral middle temporal gyrus (MTG) and inferior parietal lobule (IPL). Bilateral precuneus gyrus (PCUN) showed lower ALFF values than other regions.

**FIGURE 2:**
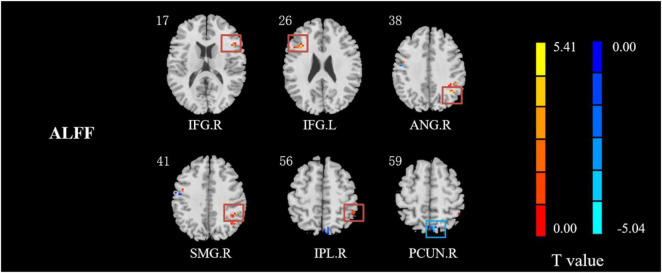
The different ALFF regions between IXT patients and HCs. ALFF values in IXT patients increased in the right ANG, SMG, IPL, and the bilateral IFG, and decreased in the right PCUN. (GRF corrected, *P* < 0.05.) ANG: angular gyrus; SMG: supramarginal gyrus; IPL: inferior parietal lobule; IFG: inferior frontal gyrus; PCUN: precuneus.

**FIGURE 3:**
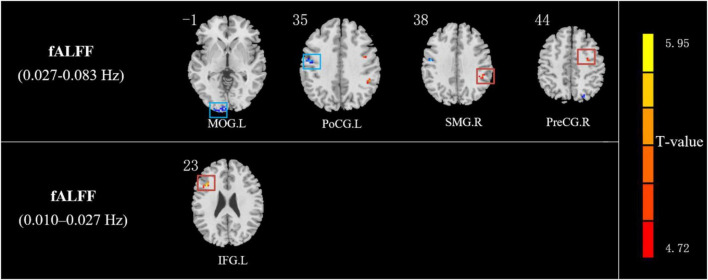
The different fALFF values of brain regions between IXT patients and HCs. The fALFF values in slow 4 (0.027–0.073 Hz) band increased in the right SMG and right PreCG and decreased in the left MOG and PoCG in IXT patients. The fALFF values in slow 5 (0.01–0.027 Hz) band increased in the left IFG in IXT patients. (GRF corrected, *P* < 0.05). SMG: supramarginal gyrus; PreCG: precentral gyrus; IFG: inferior frontal gyrus; MOG: middle occipital lobe gyrus; PoCG: postcentral gyrus.

Compared with HCs, IXT patients had significant ALFF values increase the right angular gyrus (ANG), supramarginal gyrus (SMG), IPL, the bilateral inferior frontal gyri (IFG), and a decrease in the right PCUN (Figure 2, and Table 2). Meanwhile, in the IXT group, the fALFF values were significantly increased in the right SMG and right precentral gyrus (PreCG) and decreased in the left middle occipital gyrus (MOG) and postcentral gyrus (PoCG) for the slow 4 band (Figure 3, and Table 3). Increased fALFF values were found in the left IFG for the slow 5 band (Figure 3, and Table 3).

**TABLE 2:** Regions revealing significant ALFF differences between IXT patients and HCs (*P* < 0.05, corrected for GRF).

**Brain region**	**Peak MNI, mm**			**Peak T value**	**Number of voxels**
	**x**	**y**	**z**		
R IFG	51	21	58	5.0795	17
L IFG	–36	21	24	5.2910	17
R ANG	39	–57	36	5.4563	19
R SMG	48	–42	36	5.0933	40
R IPL	48	–42	36	5.0933	40
R PCUN	6	–72	57	–4.4851	19

*R: right; L: left; MNI: Montreal Neurological Institute; IFG: inferior frontal gyrus; ANG: angular gyrus; SMG: supramarginal gyrus; IPL: inferior parietal lobule; PCUN: precuneus.*

**TABLE 3:** Regions revealing significant fALFF differences between IXT patients and HCs (*P* < 0.05, corrected for GRF).

**fALFF**	**Brain region**	**Peak MNI, mm**			**Peak T value**	**Number of voxels**
		**x**	**y**	**z**		
Slow 5 band	L IFG	–39	18	24	5.5655	14
Slow 4 band	R PreCG	36	–3	42	4.2404	14
	R SMG	48	–42	39	4.8967	14
	L PoCG	–48	–9	36	–4.6384	19
	L MOG	–27	–102	3	–4.6430	39

*R: right; L: left; MNI: Montreal Neurological Institute; IFG: inferior frontal gyrus; PreCG: precentral gyrus; SMG: supramarginal gyrus; MOG: middle occipital lobe gyrus; PoCG: postcentral gyrus.*

### Voxel-Wise Functional Connectivity Analysis

**FIGURE 4:**
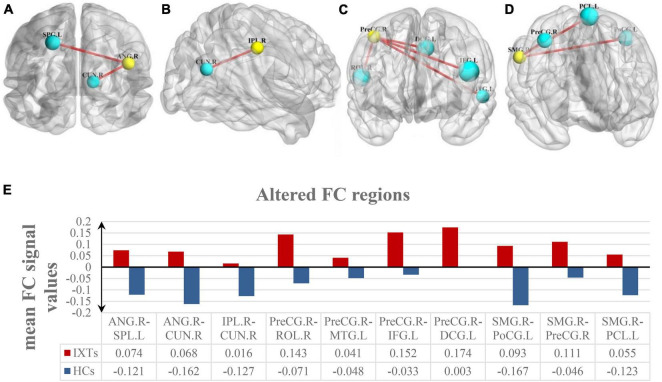
Intergroup comparison of voxel-wise FC between the IXT patient and HCs. FC between the right ANG and right CUN, left SPL was significantly increased **(A)**. FC between the right IPL and right CUN was significantly increased **(B)**. FC between the right PreCG and right ROL, left MTG, left IFG, and left DCG was significantly increased **(C)**. FC between the right SMG and left PoCG, right PreCG and left PCL was significantly increased **(D)**. The yellow balls indicate the seed ROI, while the cyan balls indicate the ROIs with significantly different FC values. The size of the cyan balls corresponds to the *t*-values from the two-sample *t*-tests. The mean FC values of altered brain region is shown in **(E)** and the details of the negative regions can be found in Table 4. ANG: angular gyrus; SPL: superior parietal lobule; CUN: cuneus; IPL: inferior parietal lobule; PreCG: precentral gyrus; ROL: rolandic operculum; MTG: middle temporal gyrus; IFG: inferior frontal gyrus; DCG: median cingulate gyrus; SMG: supramarginal gyrus; PoCG: postcentral gyrus; PCL: paracentral gyrus.

Among the intergroup comparisons, we found four ROIs with significantly increased FC with other brain clusters in the IXT group (Figure 4 and Table 4). Compared with HCs, IXT patients showed increased FC between right ANG and right cuneus (CUN), left superior parietal lobule (SPL). The FC between right IPL and right CUN were increased. The FC between right PreCG and right rolandic operculum (ROL), left MTG, left IFG, and left median cingulate gyrus (DCG) were increased. The FC between right SMG and left PoCG, right PreCG and left paracentral gyrus (PCL) was also increased. No decreased FC was observed.

**TABLE 4:** The peak MNI coordinates and intensity of brain clusters with significant intergroup differences in voxel-wise FC values (*P* < 0.05, corrected for GRF).

**ROI**	**Brain region**	**Peak MNI, mm**			**Peak T value**	**Number of voxels**
		**x**	**y**	**z**		
**R ANG**	L SPL	–24	–66	48	4.7865	23
	R CUN	12	–78	28	4.6788	14
**R IPL**	R CUN	10	–79	30	5.0084	38
**R PreCG**	R ROL	50	–2	15	5.3324	311
	L MTG	–54	–34	–2	4.0213	30
	L IFG	–54	9	15	5.3297	309
	L DCG	–7	4	46	4.7603	122
**R SMG**	L PoCG	–44	–26	59	4.7762	44
	R PreCG	9	–16	67	4.6095	82
	L PCL	–7	–29	69	5.6774	63

*R: right; L: left; MNI: Montreal Neurological Institute; ANG: angular gyrus; SPL: superior parietal lobule; CUN: cuneus; IPL: inferior parietal lobule; PreCG: precentral gyrus; ROL: rolandic operculum; MTG: middle temporal gyrus; IFG: inferior frontal gyrus; DCG: median cingulate gyrus; SMG: supramarginal gyrus; PoCG: postcentral gyrus; PCL: paracentral gyrus.*

### Correlation Between Amplitude of Low-Frequency Fluctuations/Fractional Amplitude of Low-Frequency Fluctuation, Functional Connectivity Values, and Clinical Characteristics

**FIGURE 5:**
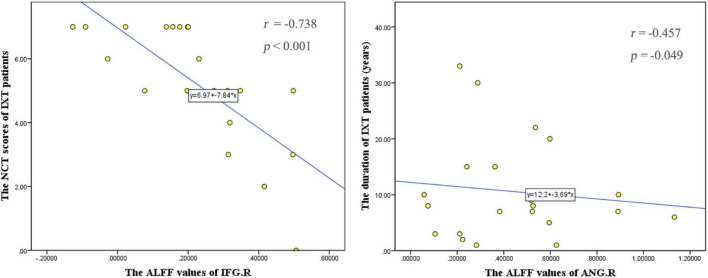
Correlations between the ALFF values and behavioral performance in IXT patients. The NCT scores were negatively correlated with ALFF values in the right IFG (*r* = –0.738, *p* < 0.001). The duration of IXT was negatively correlated with ALFF values of the right ANG (*r* = –0.457, *p* = 0.049). IFG: inferior frontal gyrus; ANG: angular gyrus.

The NCT score was negatively correlated with ALFF values in the right IFG (*r* = −0.738, *p* < 0.001). The duration of IXT was negatively correlated with ALFF values the right ANG (*r* = −0.457, *p* = 0.049) (Figure 5). There is no correlation found between fALFF and FC values and clinical characteristics.

### Validation Analysis

Global GMV is (0.697 ± 0.053) × 10^6^ mm^3^ in IXT patients and (0.678 ± 0.067) × 10^6^ mm^3^ in HCs. No significant difference was found in global GMV between groups (*t* = 1.080, *p* = 0.286). There were no clusters survived after correction for multiple comparisons in voxel-based analysis between the two groups, corrected for GRF.

After correction with GMV as an additional covariate of no interest, the brain regions with altered ALFF/fALFF values showed the same distributions to those observed without GMV correction. The results suggested that most of the ALFF/fALFF value changes were independent of GMV atrophy in IXTs.

## Discussion

Compared with HCs, the IXT patients showed significantly decreased ALFF/fALFF values in the right PCUN and increased in the bilateral IFG, the right ANG, right SMG gyrus, right IPL, right PreCG, and decreased in the left MOG, PoCG, which are associated with fusion, stereoscopic vision, and eye position control. Then we analyzed the FC of these activity-altered brain regions. Increased FC was found between these areas and other brain regions. These results prove that the neurological activity changes of IXT involve vision- and oculomotor-related brain regions. Furthermore, we found a negative correlation between the duration of IXT, the NCT scores, and ALFF values in part of the brain regions. The previous studies have suggested that the ALFF and FC could be influenced by regional GM atrophy which may lead to artificial reduction in regional ALFF and FC results (Oakes et al., 2007). In addition, ametropia may lead to changes in regulatory function and then affect the results (Kim and Kim, 2017). In this study, the differences of GMV and bilateral best-corrected VA between IXTs and HCs were not significant, which confirmed the results of ALFF and fALFF changes in IXTs are credible.

In the current study, the ALFF values of MOG were decreased. The MOG participates the stereovision function which also plays an important role in the category-selective attention-modulated unconscious face/tool processing and spatial processing (Fortin et al., 2002; Tu et al., 2013). Zhu et al. (2018) found that the FC values of MOG were significantly decreased in patients with concomitant exotropia, while IXT is a subtype of concomitant exotropia. Consistently, we demonstrated that the decreased brain function of MOG reflects the defects in the stereovision function in IXT patients. However, before IXT developed into constant exotropia, part of stereovision function still existed, suggesting that some stereopsis related brain regions might have compensatory functions. Increased ALFF/fALFF values were found in the right ANG, right SMG, and right IPL, which supported stereoscopic vision impaired in IXT. The main visual pathways include the ventral and dorsal streams (Lee et al., 2000). The dorsal visual pathway originates from the V1, passes through V2 and middle temporal areas, and arrives at the IPL. This pathway primarily takes part in the processing of spatial position information and eye movement (Merigan and Maunsell, 1993; Tootell et al., 1998). The ANG and SMG occupy a part of the IPL. The IPL extracts information about depth from motion to form stereoscopic vision, and is involved in the dynamic display of objects, providing immediate instructions to induce attentional shifts from one object to another (Yang et al., 2014). In patients with IXT, stereoscopic vision is damaged to varying degrees. To produce effective stereoscopic vision, IXT patients may require more neural activity in the dorsal visual pathway. Previous studies of strabismus and its subtype, including infantile esotropia, have found increased spontaneous activities in the ANG (Yang et al., 2014; Tan et al., 2016). A previous task-fMRI study reported that the IPL exhibited increased activation intensity in IXT patients (Li et al., 2016), which are similar to the current findings. Therefore, the increased ALFF/fALFF values of brain regions in dorsal visual pathway may be a compensatory process of stereoscopic vision.

Meanwhile, IXT patients showed increased FC between right ANG and right CUN, left SPL and the FC between right IPL and right CUN, between right SMG and left PoCG, right PreCG and left PCL was also increased. The CUN plays an important role in spatial processing, which is in charge of modifying and transmitting visual information to the extrastriate cortex (Daw, 1998). The SPL is responsible for transmitting visual information to the frontal lobe to control visuomotor integration and encode visual and proprioceptive targets, which plays a vital role in body position (Caminiti et al., 1996; Felician et al., 2004; Iacoboni and Zaidel, 2004; McGuire and Sabes, 2011). The PoCG, PreCG, and PCL belong to the motor and sensory networks, which is related to the ocular movement. The strengthening of the FC between the region of dorsal visual pathway and CUN, SPL further suggests that this is a compensatory effect for impaired stereoscopic vision.

Amplitude of low-frequency fluctuations values of the right PCUN in IXT patients were lower than those of HCs, which indicated the underlying mechanism of IXT should be defective binocular fusion proposed by many neurologists and pathologists (Pettigrew et al., 1968; Poggio and Fischer, 1977). A well-coordinated binocular fusion function is required before normal stereoscopic vision can be formed. The PCUN is crucial to visual processing, belonging to the occipital cortex and participating in both the dorsal and ventral visual pathways (Nagahama et al., 1999; Cavanna and Trimble, 2006; Tan et al., 2016; Hensch and Quinlan, 2018). Given fusion-related stimulation by task-based fMRI, Li et al., 2016 also found BOLD signals increased in the bilateral PCUN and concluded that the PCUN was associated with binocular fusion (Fu et al., 2014). Meanwhile, the PCUN is believed to play an important role in the visuomotor coordination (Nagahama et al., 1999; Cavanna and Trimble, 2006; Hensch and Quinlan, 2018). The decreased PCUN activities in patients with IXT may have caused the decreased fusion and visuomotor coordination.

In addition to the PCUN, we also found abnormal spontaneous brain activity in PreCG, PoCG, and IFG. As mentioned above, the PreCG and PoCG belong to the motor and sensory networks, which are related to the ocular movement. A previous study demonstrated that the PreCG is involved in the encoding of oculomotor (Iacoboni et al., 1997). Moreover, another research showed that stimulating the PreCG controls eye movement (Blanke et al., 2000). Zhu et al. (2018) reported that neural activity in the left PreCG was decreased in patients with comitant exotropia. The frontal lobe serves a vital role in visual processing and associated eye movements (Shao et al., 2019). It is well established that the IFG is critical for response inhibition as a part of the frontal cortex and may be involved in the suppression of eye movement (Aron et al., 2003). The lower regional homogeneity (Reho) values were observed in IFG of patients with strabismus and amblyopia (Shao et al., 2019). However, our study found increased ALFF/fALFF values in the PreCG and IFG, which is different from previous studies. We speculated IXT, as a transitional stage to constant exotropia, still has a part of function to control eye position. The increased FC was found between right PreCG and right ROL, left MTG, left IFG, and left DCG. Roker reported the activation of MTG with the fusion stimulus, and MTG is a region responsible for 3D surface orientation and retinal image velocities (Nguyenkim and DeAngelis, 2003; Rokers et al., 2009). The DCG is an important element of visual cortices, where it helps control visual attention and the encoding of visual information (Hickey et al., 2010). The increased ALFF/fALFF values in the PreCG, IFG, and increased FC between these regions and brain areas related to the ocular movement indicates a compensation and devotion to remaining in the normal eye position. Some scholars believe that ROL is associated with self-rating affective and apathetic depressive symptoms, which may indicate symptoms of depression of IXT patients (Sutoko et al., 2020).

In the IXT, abnormal spontaneous neural activities were mainly located in the right hemisphere, especially dorsal spatial processing pathway (Kwee et al., 1999; Nishida et al., 2001; Fortin et al., 2002). Consistent with our results, Yan et al. (2010) suggested that the dorsal visual pathway was abnormal or impaired in patients with comitant exotropia, combining VBM and voxel-based analysis of DTI results. Interestingly, utilizing fMRI and PET, many studies also found that the stereopsis areas were in the right parietal lobule and its surrounding areas (Kwee et al., 1999; Nishida et al., 2001). In our study, these stereopsis areas of the right hemisphere are likely to be impaired in IXT patients, leading to impaired stereopsis.

The NCT scores are the standard measure for evaluating the function of eye position control of IXT. The higher NCT scores represent more severity of IXT. The activities in the IFG were lower with increased severity of IXT in the study, which suggest decreased compensation with the progression of IXT. When IXT evolves into constant exotropia, decompensation will occur at the end, which is supported by Shao et al. They found the Reho values in IFG are lower in patients with strabismus and amblyopia (Aron et al., 2003). Furthermore, we found a negative correlation between the ALFF values of the right ANG and duration. The ANG is involved in the formation of stereoscopic vision, and with the prolongation of duration of disease, the impaired stereoscopic vision of patients is aggravated, then the activity of ANG maybe decreased.

In conclusion, changed ALFF/fALFF values and FC values are found in vision- and eye movement-related regions in IXT patients. In addition, the changes in activity of certain brain regions are associated with duration and the severity of the disease.

## Limitations

The current study involved several limitations that should be considered. First, despite us investigating the brain function changes of IXT, we did not carry on the study on the different subtypes of IXT. Second, we did not consider psychological problems of IXT patients. Meanwhile, the sample size of the study is not very large, studying a larger sample would be useful to allow more detailed neurophysiological and neuroimaging investigations. In the future, we will continue to expand the sample size and gather neuropsychological assessment data for deeper and more detailed study.

## Conclusion

The patients with IXT showed widespread ALFF/fALFF changes of neural activity and FC changes, which were located in the fusion-, stereopsis-, and oculomotor-related regions. These results indicated that the IXT is a complex central nervous system disease affecting multiple brain regions. Moreover, abnormal spontaneous neural activities in the right IFG and the right ANG are correlated with the progression of IXT, suggesting that ALFF and fALFF might be useful complementary indicators for the disease. The current findings extend understanding of the neuropathological mechanisms of IXT.

## Data Availability Statement

The raw data supporting the conclusions of this article will be made available by the authors, without undue reservation.

## Ethics Statement

This study was approved by Medical Research Ethics Committee and Institutional Review Board of Capital Medical University, Beijing Tongren Hospital. The participants provided their written informed consent to participate in this study.

## Author Contributions

XH, ZL, and JF contributed to conception and design of the study. YG, JH, and XH organized the database. XH, WL, and JL performed the statistical analysis. XH wrote the first draft of the manuscript. XH and QW wrote sections of the manuscript. XQ, TL, and LZ provide technological support. All authors contributed to manuscript revision, read, and approved the submitted version.

## Conflict of Interest

The authors declare that the research was conducted in the absence of any commercial or financial relationships that could be construed as a potential conflict of interest.

## Publisher’s Note

All claims expressed in this article are solely those of the authors and do not necessarily represent those of their affiliated organizations, or those of the publisher, the editors and the reviewers. Any product that may be evaluated in this article, or claim that may be made by its manufacturer, is not guaranteed or endorsed by the publisher.

## Footnotes

1
http://restfmri.net/forum/DPARSF

2
http://www.fil.ion.ucl.ac.uk/spm/
